# Effect of Folic Acid Supplementation on Levels of Circulating Monocyte Chemoattractant Protein-1 and the Presence of Intravascular Ultrasound Derived Virtual Histology Thin-Cap Fibroatheromas in Patients with Stable Angina Pectoris

**DOI:** 10.1371/journal.pone.0070101

**Published:** 2013-07-25

**Authors:** Kjetil H. Løland, Øyvind Bleie, Elin Strand, Per M. Ueland, Jan E. Nordrehaug, Hector M. Garcia-Garcia, Patrick W. Serruys, Ottar Nygård

**Affiliations:** 1 Department of Clinical Science, University of Bergen, Bergen, Norway; 2 Department of Heart Disease, Haukeland University Hospital, Bergen, Norway; 3 Laboratory of Clinical Biochemistry, Haukeland University Hospital, Bergen, Norway; 4 Cardialysis, Rotterdam, The Netherlands; 5 Interventional Cardiology Department, Thoraxcenter, Erasmus MC, Rotterdam, The Netherlands; King’s College London School of Medicine, United Kingdom

## Abstract

**Background:**

Virtual Histology Intravascular Ultrasound (VH–IVUS) may be used to detect early signs of unstable coronary artery disease. Monocyte Chemoattractant Protein-1 (MCP-1) is linked with coronary atherosclerosis and plaque instability and could potentially be modified by folic acid treatment.

**Methods:**

In a randomized, prospective study, 102 patients with stable angina pectoris (SAP) received percutaneous coronary intervention and established medical treatment as well as either homocysteine-lowering folic acid/vitamin B_12_ (±B_6_) or placebo (±B_6_) for 1 year before VH–IVUS was performed. The presence of VH-Thin-Cap Fibroatheroma (VH-TCFA) in non-intervened coronary vessels was registered and serum levels of MCP-1 were measured. The patients were subsequently followed for incident myocardial infarction (MI).

**Results:**

Patients treated with folic acid/vitamin B_12_ had a geometric mean (SD) MCP-1 level of 79.95 (1.49) versus 86.00 (1.43) pg/mL for patients receiving placebo (p-value 0.34). VH-TCFA lesions were present in 7.8% of patients and did not differ between intervention arms (p-value 0.47). Serum levels of MCP-1 were 1.46 (95% CI 1.12 to 1.92) times higher in patients with VH-TCFA lesions than in those without (p-value 0.005). Afterwards, patients were followed for median 2.1 years and 3.8% experienced a myocardial infarction (MI), which in post-hoc Cox regression analyses was independently predicted by both MCP-1 (P-value 0.006) and VH-TCFA (p-value 0.01).

**Conclusions:**

In patients with SAP receiving established medical treatment, folic acid supplementation is not associated with either presence of VH-TCFA or levels of MCP-1. MCP-1 is however associated with VH-TCFA, a finding corroborated by increased risk for future MI.

**ClinicalTrials.gov Identifier: NCT00354081.**

## Introduction

While significant progress has been made in both diagnosis and treatment of coronary artery disease (CAD), a substantial number of patients still experience recurrent coronary events [1]. Common underlying features of these events are non-flow-limiting, angiographically non-significant stenosis – culprit lesions with an unstable atherosclerotic plaque phenotype. Morphologically, these lesions are characterized by: a thin cap fibrous atheroma (TCFA) overlying a necrotic core with an extensive inflammatory infiltrate, a plaque erosion or a calcified nodule [2-4]. In recent years, it has been increasingly apparent that such lesions are not satisfactorily diagnosed in clinical practice, and there is a growing agreement that early detection and intensive treatment of vulnerable lesions are paramount in order to decrease the rate of coronary events in patients with medically treated stable angina pectoris (SAP) [5].

One well-developed technology applicable for this setting is spectral analysis of intravascular ultrasound (IVUS) radio frequency data [6] called Virtual Histology IVUS (VH-IVUS). Using VH–IVUS one can identify VH-Thin-Cap Fibroatheroma (VH-TCFA). VH-TCFA has been reported to predict clinical events in prospective studies [7] and identification of patients at-risk is feasible if one could identify occult VH-TCFA lesion.

The recognition of the inflammatory response as playing a pivotal role in the formation and progression of atherosclerosis has been one of the leading breakthroughs in recent years [8]. High-sensitivity C-reactive protein (hsCRP) has already earned its mark as an independent risk factor [9] and several other inflammatory markers have been proposed. Nevertheless, despite optimal pharmacological treatment and revascularization, a sub-group of patients continue to experience cardiovascular events that are thought to be the end-point of an inflammatory process not adequately controlled by conventional treatment.

Monocyte Chemoattractant Protein-1 (MCP-1), also known as Chemokine ligand [C-C motif] 2 (CCL2), is an extensively studied chemokine involved in the recruitment of monocytes, dendritic cells and T_h_-cells [10] and has been associated to macrophage activity in atherosclerotic plaques [11-13]. Hyperhomocysteinemia has been linked with elevated MCP-1 [14] and it has been shown that folic acid supplementation lowers the levels of MCP-1 in hyperhomocysteinemic rats [15]. Interestingly, studies in humans have shown that folic acid supplementation lowers levels of MCP-1 in obese patients [16]. In children with low body mass index (BMI) there is a negative correlation between MCP-1 and plasma homocysteine which is not present in obese children [17].

Hyperhomocysteinemia has been associated with cardiovascular disease in several prospective studies [18] and numerous explanatory pathomechanisms have been proposed [19-21]. Treatment with B-vitamins, including folic acid and vitamin B_12_, has been shown to lower plasma homocysteine by supplying methyl groups for remethylation of homocysteine. More than 20 years of homocysteine studies have culminated in several large scale clinical intervention trials that have shown no effect on cardiovascular mortality from homocysteine-lowering folic acid supplementation [20,22-26].

The goal of the current study was to explore the effect of folic acid supplementation on the levels of MCP-1 and presence of VH-TCFA in a cohort of patients with established CAD and optimally treated SAP and further to investigate the relationship between MCP-1 and VH-TCFA.

## Methods

### Study design and patient population

This trial was a sub-study of the Western Norway B-vitamin Intervention Trial (WENBIT) in collaboration with the Global Virtual Histology Intravascular Ultrasound (VH–IVUS) Registry. WENBIT investigated the effect of homocysteine-lowering B-vitamin supplementation on cardiovascular end-points as secondary prevention in a non-fortified population. The patients were randomized and given B-vitamin supplementation according to a 2x2 factorial design previously described [27]; receiving an oral capsule with one of the following compositions: folic acid 0.8 mg plus vitamin B_12_ (cyanocobalamin) 0.4 mg with or without vitamin B_6_ (pyridoxine) 40 mg or vitamin B_6_ alone or placebo (defined as WENBIT-baseline).

**Figure 1:**
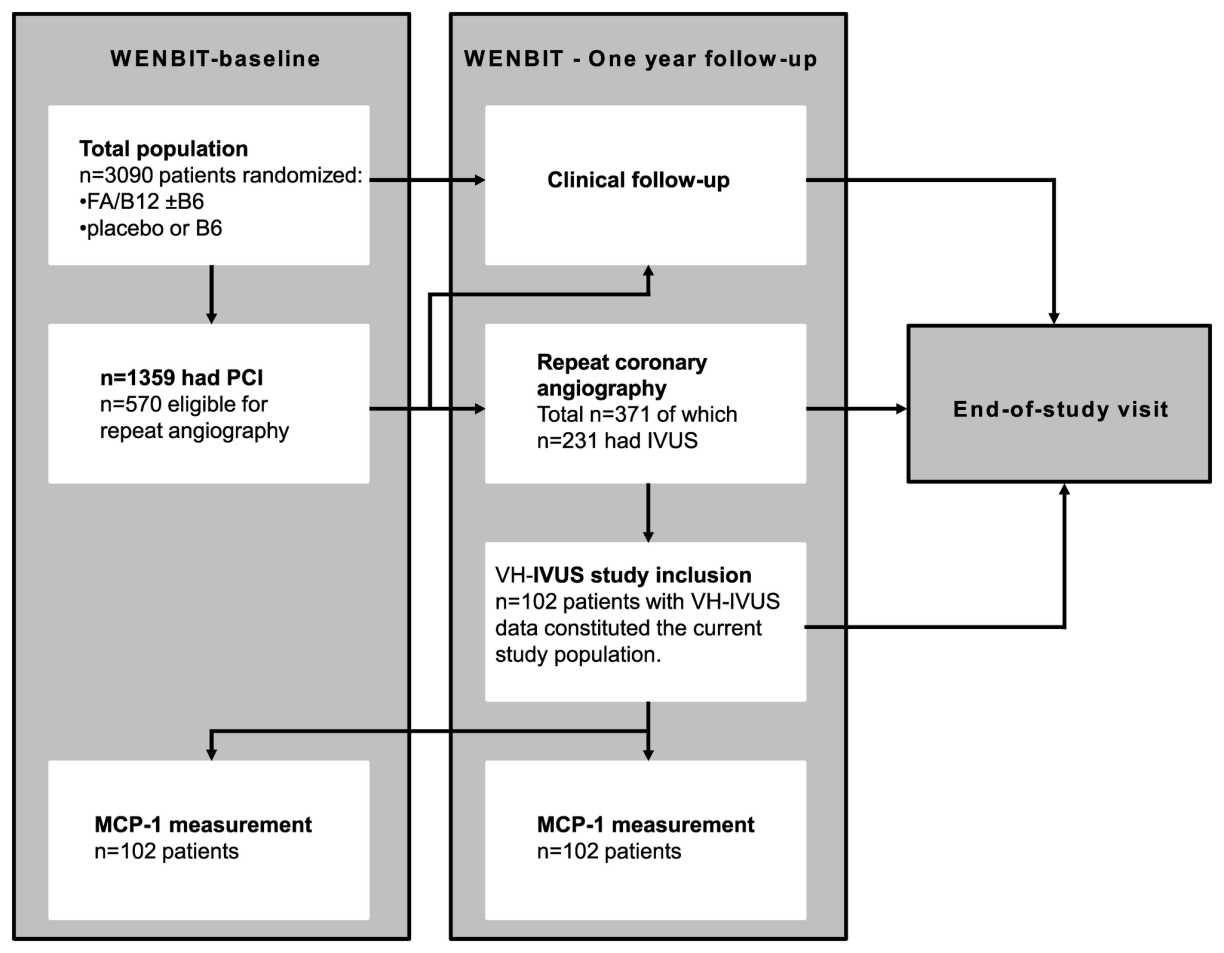
Flow-chart of patient inclusion. The chart shows the flow of patients from the WENBIT-trial (n=3090) to inclusion in the current IVUS-VH study (n=105). All patients were randomized to B-vitamin treatment at WENBIT-baseline. Of the 1359 patients who had PCI at WENBIT-baseline, 371 had new, scheduled angiography at the WENBIT-one year follow-up. Of these 371 patients, 231 had received IVUS in a non-intervened vessel. IVUS-VH analysis and MCP-1 measurements were performed on 102 of these 231 patients and constituted our current study population. MCP-1 measurements were performed both at WENBIT-baseline (B-vitamin randomization) and at IVUS-VH study inclusion. Abbreviations are FA, folic acid (0.8 mg); B12, vitamin B_12_ (0.4 mg); B6, vitamin B6 (40 mg); WENBIT, Western Norway B-vitamin Intervention Trial; IVUS, intravascular ultrasound; IVUS-VH, intravascular ultrasound – virtual histology; PCI, Percutaneous coronary intervention and MCP-1, monocyte chemoattractant protein-1.

The current cohort of 105 patients was mostly recruited at the one-year follow-up visit at Haukeland University Hospital in Bergen, Norway (defined as VH–IVUS study inclusion, median [IQR] 385 [399] days after WENBIT-baseline). All study participants were recruited from a subset of patients who after percutaneous coronary intervention (PCI) at WENBIT-baseline had been scheduled for a repeat angiography after one year. Accordingly, all patients had CAD and had been treated with established medication as well as B-vitamins or placebo for at least one year at the time of VH–IVUS study inclusion. This recruitment took place from June 2004 to September 2005 and is illustrated by a flow-chart in Figure 1.

IVUS-measurements were performed by the same interventional cardiologist after intracoronary administration of nitroglycerin 0.2 mg. A 20 MHz, 2.9Fr IVUS catheter (Eagle Eye, Volcano Corp., Rancho Cordova, California) was advanced to the middle or distal segment (depending on coronary anatomy) of the selected study vessels and a motorized pullback (0.5 mm/s) was performed until the proximal arterial ostium. A study vessel was defined as non-intervened, proximal segments of PCI treated study vessels or a non-obstructive coronary study vessel technically accessible for IVUS-measurements (belonging to the left anterior descending artery, right coronary artery or circumflex artery) as previously specified [28].

VH–IVUS data was lost in three patients due to corrupted data on disc, i.e. 102 patients had complete VH–IVUS data.

### Ethics statement

All clinical investigation was conducted according to the principles expressed in the Declaration of Helsinki. Written informed consent was obtained from all WENBIT participants, and an additional written informed consent was collected from patients included in the current VH–IVUS sub-study. Both WENBIT and the current sub-study were approved by the Regional Committee for Medical and Health Research Ethics (Regional Ethics Committee [REC] West/Regional Etisk Komité [REK] vest, which is the institutional review board available at http://helseforskning.etikkom.no), the Norwegian Medicines Agency (Legemiddelverket) and by the Data Inspectorate (Datatilsynet). WENBIT was registered with ClinicalTrials.gov with identifier NCT00354081 and URL http://www.clinicaltrials.gov/ct2/show/NCT00354081. WENBIT was a two-centre study conducted at Haukeland University Hospital, Bergen, and Stavanger University Hospital, Stavanger, both located in western Norway. All clinical research was conducted in Norway and was subject to Norwegian law.

### IVUS, VH-imaging and end-point definition

All IVUS-data were transferred to the Global VH–IVUS Registry Cardialysis, BV core lab for analyses using the pcVH 2.2 software (Volcano corp., Brussels, Belgium). This is a validated method for visualizing plaque morphology *in vivo*. IVUS-VH renders four different histological tissue components, namely fibrous, fibro-fatty, necrotic core and calcified, all with high correlation with histopathology in *ex vivo* validation studies [3,6,29]. Each of these neointimal components where presented as total volume (mm^3^) and percentage volume of total plaque volume. An IVUS-lesion is present when the plaque burden (plaque volume / external elastic membrane volume) exceeds 40% over three consecutive frames. A VH-TCFA lesion was present when a necrotic core rich (>10% of cross-sectional area) element was identified in at least 3 consecutive frames with an arch of necrotic core in contact with the lumen for at least 36° of the circumference, as per standardized IVUS image acquisition [30]. While pull-back was done in a per-vessel manner, data from the core lab was provided in a per-lesion form. For subsequent analyses lesion-level data was compressed to and presented as patient-level data (i.e. VH-TCFA is presented as VH-TCFA per patient and not per coronary lesion).

### Clinical WENBIT end-of-study follow-up

Patients were followed from VH–IVUS study inclusion for median (range) 734 (42–920) days (defined as WENBIT end-of-study visit). The follow-up endpoint was defined as a composite of fatal and non-fatal MI. All events were adjudicated by the WENBIT-committee, unbeknownst of the MCP-1 serum levels and VH-TCFA status of the patient.

### Blood samples

Blood samples were collected at study visits. Routine blood analyses such as hematologic parameters, renal function markers and lipid-related factors were analyzed in fresh samples at the Laboratory of Clinical Biochemistry, Haukeland University Hospital, by standard methods. Blood samples for measurements of MCP-1 were analyzed at the Institute of Medicine in collaboration with the Lipid Group, University of Bergen, Norway using BioPlex^®^ 200 multiplex array (Bio-Rad Laboratories, Hercules, CA, US). Blood samples for measurements of B-vitamins and total homocysteine (tHcy) were analyzed at the laboratory of Bevital AS (www.bevital.no), Bergen, Norway.

### Statistical analysis

All analyses was done using the *R* software version 2.12-15.1 [31]. Figures were made using the R package *ggplot2*, version 0.8.3.Variables are presented as either mean (±SD) or median (interquartile range) for continuous variables and count (%) for categorical variables. Differences in clinical and biochemical characteristics at WENBIT-baseline and VH–IVUS study inclusion between intervention groups or VH-TCFA groups were analyzed with Welch two-sample t-test or chi-square/Fisher’s exact test with minimum likelihood where appropriate. Change in MCP-1 levels from WENBIT-baseline to VH–IVUS study inclusion for the whole group was done using Welch two-sample t-test of log-transformed data. Geometric mean and SD are presented. The effect of folic acid supplementation levels on MCP-1, measured at both WENBIT-baseline and VH–IVUS study inclusion, was evaluated with Welch two-sample t-test as well as with a multivariate generalized additive model (GAM) adjusted for age, gender, BMI, creatinine, C-reactive protein, apolipoprotein B100, total homocysteine, systolic blood pressure, diabetes, smoking and statin use. The association of VH-TCFA presence and folic acid supplementation was analyzed with Fisher’s exact test with minimum likelihood as well as an extended generalized additive model adjusting for age, gender, BMI, creatinine, C-reactive protein, apolipoprotein B100, tHcy, systolic blood pressure, diabetes, smoking and statin use. For skewed variables we applied logarithmic transformation in order to approximate normal distribution except for GAM analyses. Back-transformation was applied for data presentation.

A post-hoc, hypothesis-generating evaluation of the putative prognostic effect of VH-TCFA on ensuing risk of myocardial infarction (MI) during subsequent follow-up as well as relationship of VH-TCFA with MCP-1 was done. Association between MCP-1 levels and MI was analyzed by chi-square test and multivariate logistic regression adjusting for age, gender, smoking status, diabetes, systolic blood pressure, BMI, hsCRP and apolipoprotein B-100. Time to MI was evaluated using additive Cox Proportional Hazard models. MCP-1 at VH–IVUS study inclusion was entered as continuous variable in crude, exploratory analyses. Multivariate models included adjustment for age, gender, hypertension, smoking status, diabetes mellitus, creatinine, hsCRP and apolipoprotein B-100.

Multiple imputation using a bootstrapping-based algorithm [32] was applied to deal with missing data. Missing data included apolipoprotein A-1, apolipoprotein B-100, systolic and diastolic blood pressure, all n=1, but not from the same patient. A two-sided p-value of < 0.05 was considered statistically significant.

## Results

### Patient characteristics

Clinical characteristics at the time of randomization for folic acid/vitamin B_12_ treatment at WENBIT-baseline is shown in table 1. Of the 105 patients, 55 (53.9%) received folic acid and vitamin B_12_ intervention, with or without vitamin B_6_. At VH–IVUS study inclusion 85% of the participants were male; mean (SD) age was 60.7 (9.7) years, systolic blood pressure 145.7 (20.2) mmHg, BMI 27.0 (2.9) kg/m^2^ and ejection fraction 63.2 (7.2)%. Diabetes mellitus was present in 11%, hypercholesterolemia in 57% and 21% were current smokers. Statins were used by 98% of the population, while acetylsalicylic acid was taken by 94%.

**Table 1:** Characteristics and Laboratory Findings in All Patients (n=102) at the Time of WENBIT-baseline.

Characteristics		B-vitamin treatment		
**Demographic characteristics**		**FA/B12±B6 (n=55)**	**Placebo or B6 (n=47)**	**p-value**
	Age, years	60.0 (12.5)	61.0 (14.5)	0.86
	Male sex, no. (%)	46 (83.6)	39 (78.7)	0.86
**Clinical Characteristics**				
	Systolic blood pressure, mmHg	139.0 (29.0)	146.0 (27.5)	0.71
	Body mass index, kg/m^2^	27.0 (3.0)	27.0 (4.5)	0.41
**Cardiovascular Risk Factors, no. (%)**				
	Peripheral vascular disease	1 (1.8)	2 (4.3)	0.59
	Cerebrovascular disease	3 (5.5)	3 (6.4)	1.00
	Prior myocardial infarction	20 (36.4)	16 (34.0)	0.97
	Prior surgical revascularization	1 (1.8)	1 (2.1)	1.00
	Hypercholesterolemia*	27 (50.0)	29 (65.9)	0.17
	Hypertension**	22 (40.0)	19 (40.4)	0.87
	Diabetes mellitus I or II	6 (10.9)	5 (10.6)	0.78
	Current smoker	12 (21.8)	9 (19.1)	0.93
	Family history***	20 (36.4)	17 (36.2)	0.85
**Medications, n (%)**				
	Statins***	55 (100.0)	46 (96.9)	0.94
	β-adrenergic receptor antagonists	41 (74.5)	32 (66.0)	0.62
	Calcium antagonists	9 (16.4)	8 (17.0)	0.86
	ACE-inhibitors or ARB	18 (32.7)	15 (31.9)	0.90
	Acetylsalicylic acid	51 (92.7)	43 (91.5)	1.00
	ADP receptor antagonists	11 (20.0)	6 (14.6)	0.48
**Laboratory Findings, mean (SD)**				
	C-reactive protein, g/dL	1.06 (2.02)	1.22 (2.18)	0.81
	Hemoglobin, g/dL	14.5 (1.4)	14.1 (1.3)	0.09
	Glucose, mmol/L	6.0 (1.5)	6.0 (1.5)	0.69
	Creatinine, µmol/L	79 (15)	79 (13.5)	0.31
	Total cholesterol, mmol/L	4.3 (1.1)	4.2 (1.0)	0.86
	High density lipoprotein cholesterol, mmol/L	1.4 (0.4)	1.3 (0.4)	0.51
	Low density lipoprotein cholesterol, mmol/L	2.6 (1.0)	2.5 (0.8)	0.78
	Triglycerides, mmol/L	1.37 (0.73)	1.50 (0.59)	0.22
	Apolipoprotein B-100, g/L	0.76 (0.26)	0.75 (0.18)	0.99
	Apolipoprotein A-1, g/L	1.40 (0.23)	1.34 (0.30)	0.42
	Total plasma homocysteine, µmol/L	9.97 (3.83)	9.19 (3.21)	0.17
	Serum folate, nmol/L	11.0 (6.6)	10.2 (7.8)	0.95
	S-cyanocobalamin, pmol/L	316.2 (132.0)	326.6 (136.1)	0.24
	Monocyte chemoattractant protein-1, p g/mL	71.5 (36.8)	82.2 (30.2)	0.17

For continuous variables, median and interquartile range is presented. For categorical variables, number and percentage is presented. FA, folic acid 0.8 mg; B12, vitamin B12 0.4 mg; B6, vitamin B6 40 mg; ACE, angiotensin converting enzyme; ARB, angiotensin II receptor blocker; ADP, adenosine diphosphate; LDL, low-density lipoprotein; HDL, high-density lipoprotein. Comparison between groups was done with Wilcoxon rank sum test for continuous data and Pearson’s Chi squared test or Fisher’s exact test were appropriate for categorical data. A p-value of < 0.05 was defined as significant.

* Defined as a history of untreated total serum cholesterol > 6.5 mmol/L or familial hypercholesterolemia. 4 patients with missing data.

** Defined as systolic blood pressure > 140 and/or diastolic > 90 mmHg and/or antihypertensive therapy.

*** Family history of coronary artery disease in first degree male relative before the age of 55 and before 65 in first degree female relatives.

**Figure 2:**
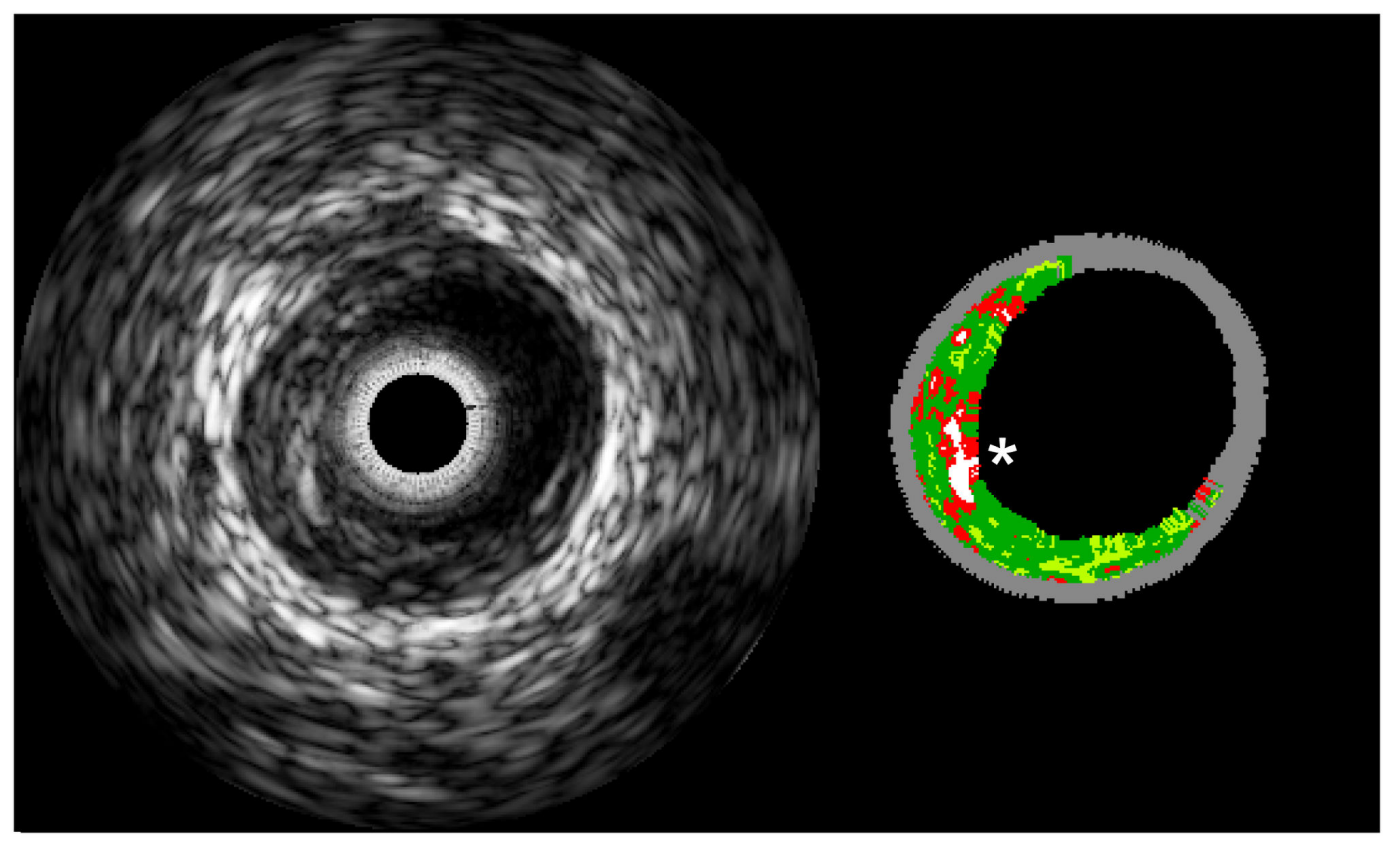
Grayscale IVUS and Virtual Histology of TCFA. A thin cap fibroatheroma (asterisk) in the proximal left anterior descending coronary artery of one of the study patients. The left panel shows an intravascular ultrasound radiofrequency image which is subsequently used to make the virtual histology image in the right panel. Green is fibrous tissue, yellow is fibro-fatty, red is necrotic core and white is calcified tissue.

IVUS-lesion characteristics at the time of VH–IVUS study inclusion are presented in table 2 and an example VH–IVUS image in Figure 2. Eight patients (7.8%) were identified with a VH-TCFA lesion at VH–IVUS study inclusion. Regarding clinical and biochemical characteristics, only apolipoprotein A1 differed statistically significant between the TCFA and non-TCFA group (p-value 0.05) when not adjusted for multiple comparisons. After the VH–IVUS study inclusion the 105 patients were followed for a median (IQR) of 734 (112) days until the end of WENBIT.

**Table 2:** Intravascular Ultrasound Derived Virtual Histology characteristics (n=102).

Variable	**Non-VH-TCFA (n=94)**	**VH-TCFA (n=8)**	**P-value**
Fibrous volume, mm^3^	70.5 (45.6)	131.7 (51.5)	< 0.01
Fibrolipidic volume, mm^3^	26.6 (24.1)	47.0 (29.6)	0.03
Necrotic core volume, mm^3^	15.9 (14.3)	29.1 (19.9)	0.02
Calcified volume, mm^3^	9.0 (6.6)	13.2 (8.2)	0.09
Relative fibrous volume, %	58 (7)	61 (7)	0.41
Relative fibrolipidic volume, %	21 (9)	20 (6)	0.81
Relative necrotic core volume, %	13 (7)	13 (6)	0.91
Relative calcified volume, %	8 (6)	6 (3)	0.70
Lesion length, mm	43.7 (18.6)	54.4 (16.8)	0.15

Categorical variables are presented as number and percentage. Absolute volume measurements are presented as mean (SD) mm3 while relative volume measurements are presented as percentage (SD). Comparison between groups were done using Wilcoxon rank sum test. A significance level of 0.05 was used. VH-TCFA, virtual histology-thin cap fibroatheroma.

### Blood indices at WENBIT-baseline and VH–IVUS study inclusion

Serum median (IQR) MCP-1 levels in the VH–IVUS study group (n=105) at WENBIT-baseline was 77.1 (34.7) and 85.3 (36.6) pg/mL at VH–IVUS study inclusion. Change in mean serum MCP-1 from WENBIT-baseline to VH–IVUS study inclusion was not significant (p=0.19). At WENBIT-baseline, median (IQR) plasma tHcy was 9.5 (3.4) µmol/L, plasma folate 10.9 (6.8) nmol/L and cyanocobalamin 321 (135) pmol/L. Folic acid/vitamin B12 supplementation significantly raised plasma folate by mean (SE) 63.7 (3.8) nmol/L (p-value <0.0001) and cobalamin by 191.3 (36.3) pmol/L (p-value <0.001). Homocysteine was lowered 29.5% with 2.8 (0.4) µmol/L (p-value <0.0001).

### MCP-1 levels according to supplementation with folic acid/B12

**Figure 3:**
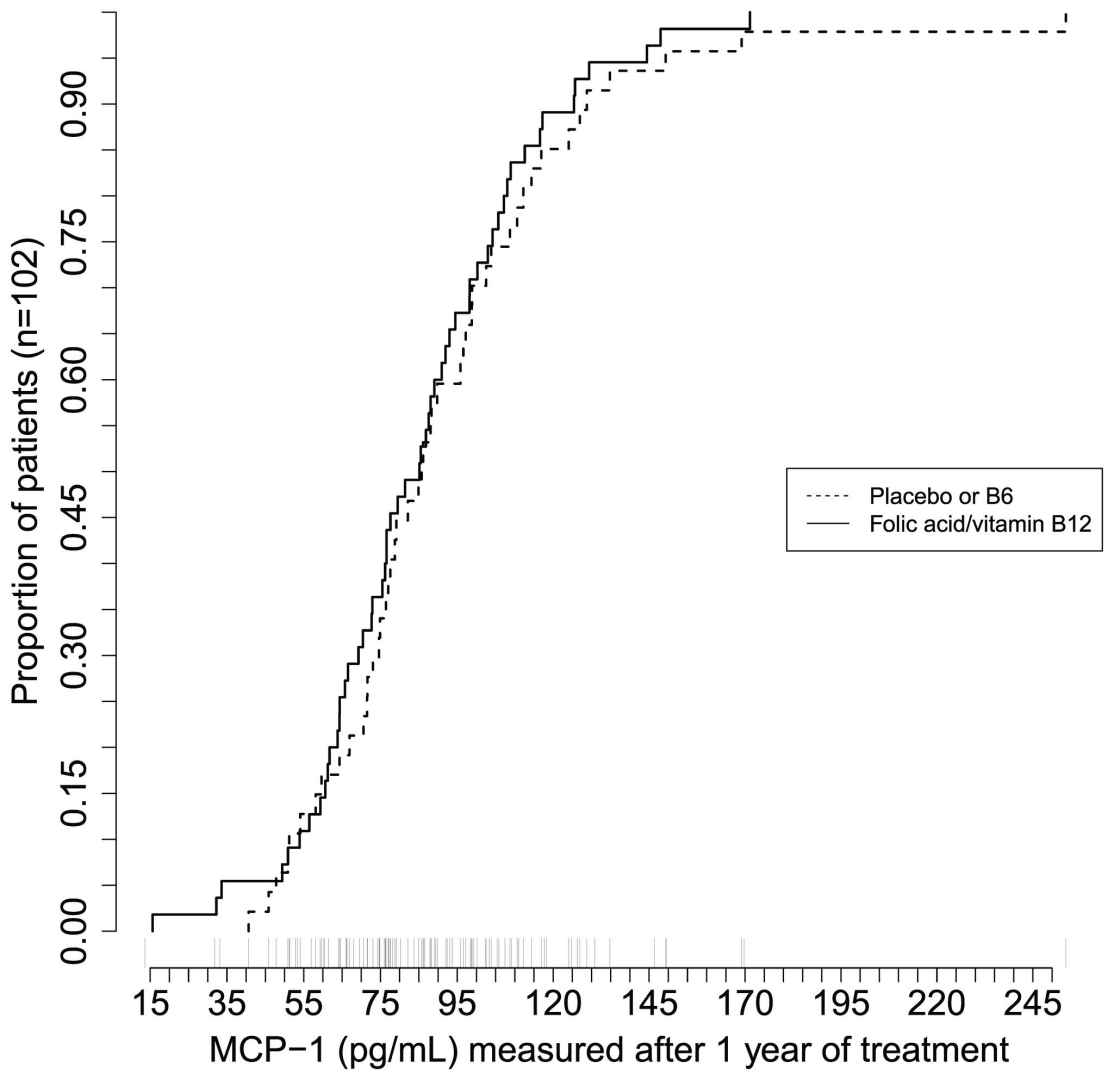
MCP-1 Levels According to Folic Acid Supplementation at VH–IVUS Study Inclusion. Cumulative distribution frequency plots showing plasma Monocyte Chemotactic Protein-1 (MCP-1) on the x-axis in patients receiving folic acid/vitamin B_12_ (solid line) and placebo or B_6_ (dashed line).

Folic acid supplementation did not result in a statistically significant difference in MCP-1 levels between the intervention groups. At WENBIT-baseline, patients receiving folic acid and vitamin B_12_ supplementation had geometric mean (SD) serum MCP-1 levels of 73.9 (1.4) pg/mL versus 81.6 (1.4) pg/mL for patients given placebo (p-value = 0.15). At VH–IVUS study inclusion patients with folic acid/vitamin B_12_ supplementation had a geometric mean (SD) MCP-1 level of 80.0 (1.5) versus 86.0 (1.4) pg/mL for patients receiving placebo (p-value 0.34) as illustrated in Figure 3. Multivariate GAM gave similar non-significant effects on MCP-1 concentration at both WENBIT-baseline and VH–IVUS study inclusion.

### VH–IVUS analyses

A total of 151 coronary vessels from the n=102 patients were analyzed yielding n=167 coronary IVUS-lesions. Analyzed coronary vessels in the 102 patients is shown in table 2 and were n=1 left main stem (0.7%), n=68 left anterior descending artery (45.0%), n=42 left circumflex artery (27.8%) and n=40 right coronary artery (26.5%). Total plaque volumes in the analyzed segments were dominated by fibrous histology. At the lesion level, fibrous histology constituted a relative volume of 58% in non-VH-TCFA lesions and 61% in VH-TCFA lesions, similarly fibro-fatty constituted 21% and 20%, necrotic volume 13% and 13% while calcified volume made up 8% and 6%. The mean IVUS-lesion length did not differ significantly between non-VH-TCFA lesions (43.7 mm) and VH-TCFA lesions (54.4 mm). A VH-TCFA lesion was identified in 7.8% of the patients. VH-TCFA lesions had significantly higher absolute volumes of both fibrous, fibrolipidic and necrotic tissue than non-VH-TCFA lesions (all p-value <0.05). The volume of calcified tissue did not significantly differ between VH-TCFA lesion and non-VH-TCFA lesions (table 2).

### VH-TCFA presence, MCP-1 levels and folic acid supplementation

**Figure 4:**
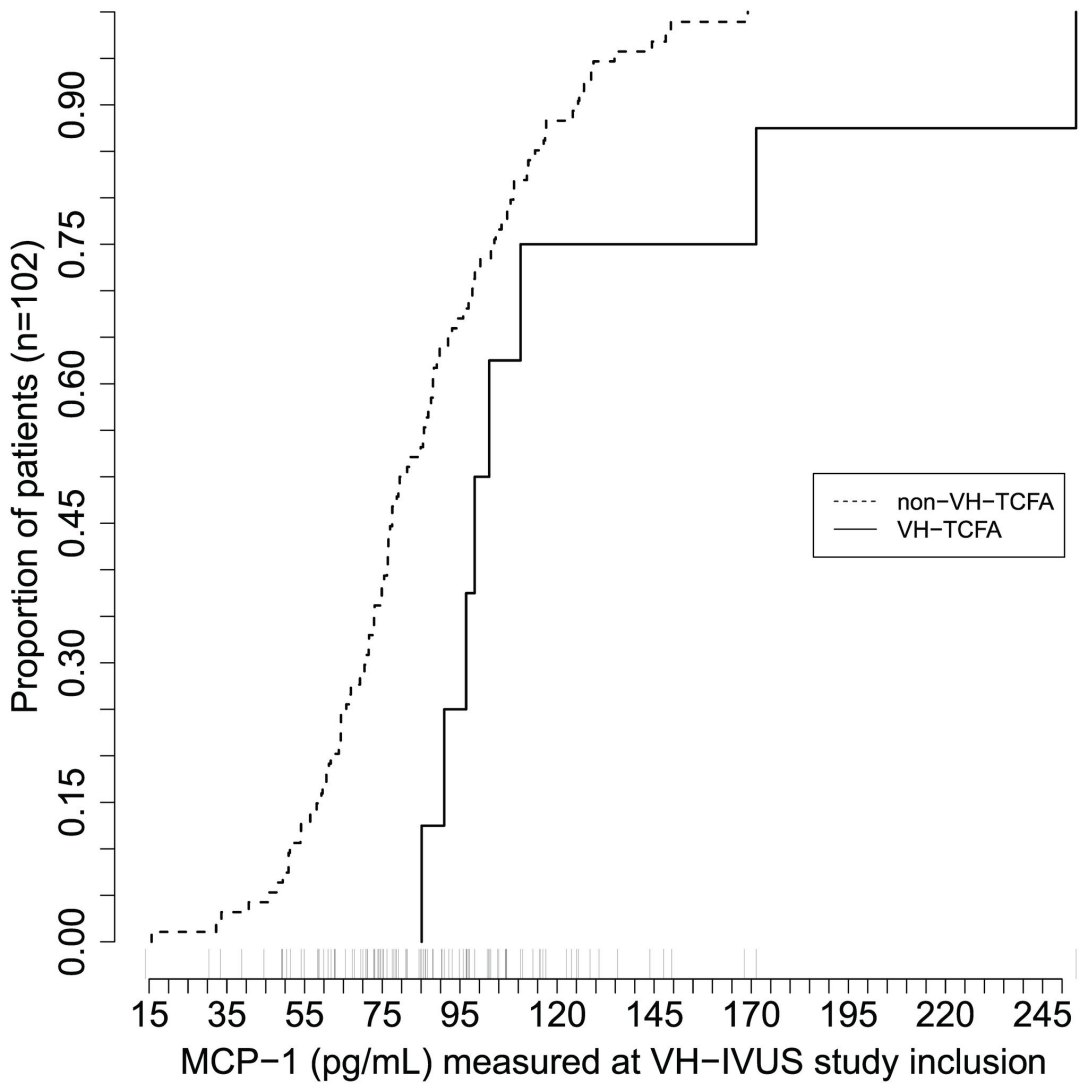
MCP-1 and Presence of Occult Thin-Cap Fibroatheroma at VH–IVUS Study Inclusion. Cumulative distribution frequency plots of Monocyte Chemotactic Protein-1 (MCP-1) in patients with (solid line) and without (dashed line) Virtual Histology Thin-Cap Fibroatheroma (VH-TCFA).

The difference in MCP-1 between VH-TCFA and non-VH-TCFA patients at VH–IVUS inclusion is shown in Figure 4 (n=102). Patients presenting with VH-TCFA lesions had a geometric mean (SD) MCP-1 level of 117.5 (1.5) versus 80.3 (1.4) pg/mL in the non-VH-TCFA patients. MCP-1 levels were 1.46 (95% CI 1.12–1.92) times higher in patients with VH-TCFA than those without (p-value 0.006). This was unaffected by multivariate adjustment (p-value 0.0006).

There was no statistical significant effect of folic acid/vitamin B_12_ treatment on the presence of VH-TCFA lesions (p-value 0.47).

### Clinical end-points

A statistically significant higher proportion of patients in the VH-TCFA group experienced an MI during the 2.1 years of follow-up between VH–IVUS study inclusion and WENBIT end-of-study visit compared to those without a VH-TCFA lesion (data not shown).

Patients with an MI during follow-up had 1.6 (95% CI 1.1 to 2.4) times higher levels of MCP-1 at VH–IVUS study inclusion than event-free patients (p-value 0.01), unaltered by multivariate adjustment (p-value 0.03). In a Cox proportional hazard model with MCP-1 levels at VH–IVUS study inclusion, hazard ratio (HR) [95% CI] for MI increased with 2.1 [0.6 to 3.6]% per pg/mL MCP-1 (p-value 0.006). In multivariate analysis the HR [95% CI] for MI for MCP-1 was 3.6 [0.5 to 6.8]% per pg/mL increase (p-value 0.02). 

## Discussion

### Principal findings

In patients with CAD receiving established medical treatment and revascularization with PCI, we found no statistically significant association between treatment with folic acid/vitamin B_12_ and either levels of MCP-1 or the presence of occult VH-TCFA lesions by IVUS. We did however find a strong, statistically significant relationship between VH-TCFA lesions and elevated serum levels of MCP-1. Additionally, in a post-hoc analysis, MCP-1 levels predicted time to MI in a Cox proportional hazard model with and without adjustment for established risk factors.

### MCP-1 levels and VH-TCFA presence according to folic acid/vitamin B_12_ treatment

While previous studies have found both an association between plasma tHcy and MCP-1 [16,33] as well as an MCP-1 lowering effect of folic acid supplementation [15,16], we were unable to reproduce these results in this cohort of patients with SAP receiving established medical therapy. While others have reported a correlation of 0.31 between tHcy and MCP-1 [33], this was not apparent amongst our patients (spearman 0.006), an observation that is corroborated by no effect of B-vitamin treatment on the presence of VH-TCFA lesions. These apparent discrepancies between results obtained by us and others [15,16] may reflect different study design and subjects characteristics, including low tHcy at WENBIT-baseline (<10 µmol/L), the presence of established CAD and statin treatment in our study population. Notably, it has been shown that in the presence of obesity and insulin resistance, the correlation between MCP-1 and tHcy is abolished [17]. Stratifying according to BMI did however not result in significant effects of folic acid/vitamin B_12_ treatment on MCP-1 levels in our study (data not shown).

### MCP-1 and plaque vulnerability

MCP-1/CCL2 is associated with coronary risk factors in sub-clinical atherosclerosis [34] and is a predictor of cardiovascular mortality in middle-aged obese patients [35]. Interestingly, MCP-1 has shown to independently predict clinical end-points in acute coronary syndromes (ACS) both in the acute [36,37] and chronic phase [37,38]. We confirmed the correlation between MCP-1 and coronary events, suggesting that this effect could be mediated through the presence of vulnerable plaques represented by VH-TCFA lesions.

Data suggest that TCFA-lesions are present in as much as two-thirds of culprit lesions [39-41]. Monocyte infiltration is a prerequisite for the development of atherosclerotic lesions, and MCP-1 plays an essential role [11-13]. MCP-1 levels and mRNA expression have recently been associated with acute coronary syndrome and plaque rupture [42]. We found that MCP-1 was correlated to VH-TCFA lesions and independently predicted MI, and 25% of patients in our study with VH-TCFA experienced a coronary event within 2 years.

MCP-1 is instrumental to the recruitment of monocytes from the bloodstream into atherosclerotic plaques [10]. In basic research, MCP-1 has been linked to macrophage activity in atherosclerotic plaques [11,12], a *sine qua non* of plaque destabilization [43,44]. Raised circulating levels of MCP-1 might represent increased monocyte recruitment, a necessary step on the ladder towards plaque rupture.

### Study limitations

There are a few study limitations to be addressed. Most important is the limited number of patients in the present study. Performing survival analysis on such data was done as post-hoc analyses and should be viewed as purely hypothesis-generating. The ability to independently predict both future MI and the anatomical substrate for that event is, however, intriguing and warranted reporting. Furthermore, the statistical models are fairly robust in spite of low number of events and were not attenuated by multivariate adjustment. A second limitation is that our patients had a relatively low-number of VH-TCFA lesions. This could possibly reflect that the patient cohort was well-treated and clinically stable. A “true” SAP-cohort is however the sub-group of interest, since this is the patient group where improved risk prediction would be interesting. Additionally, all of our patients had undergone PCI, rendering at least one coronary segment per patient unavailable for VH-TCFA evaluation, since we would have been unable to discern whether plaque instability was a result from mechanical stress due to adjacent stent deployment or inflammatory activity. Another problem with IVUS-measurements is the size of the coronary catheter, restricting analyses to proximally located lesions of large epicardially located arteries. However, a majority of ruptured plaques and TCFAs were located within the proximal segments of the left anterior descending artery and left circumflex artery in patients with cardiac death [45]. Lastly, the relatively low levels of tHcy (<10 µmol/L at baseline may have masked any anti-inflammatory effect of folic acid/vitamin B_12_ treatment.

### Study strengths

The study’s main strength is our ability to simultaneously evaluate the effect of the folic acid/vitamin B_12_ treatment on both biochemical and anatomical substrate of coronary atherosclerosis. In addition, we were able, albeit in post-hoc analyses, to demonstrate a relationship between a biomarker, the putative anatomical substrate of disease and clinical end-points. This contextualizes the MCP-1/VH-TCFA-association.

## Conclusions

In this prospective study on patients with established CAD, folic acid/vitamin B_12_ supplementation did not alter either MCP-1 levels or the presence of occult VH-TCFA coronary lesions in SAP patients. However, MCP-1 was associated with the presence of VH-TCFA lesions. In post-hoc analyses both MCP-1 and VH-TCFA independently predicted MI, possibly reflecting sustained monocytic infiltration and activity in proximally located, occult thin-cap fibroatheromas not attenuated by moderate to aggressive pharmacological treatment. This suggests that plasma levels of MCP-1 could be used to select SAP patients for further investigations, including VH–IVUS, to identify patients with VH-TCFA lesions. Further studies should aim to confirm the association between vulnerable plaques and levels of MCP-1 and whether MCP-1 measurement could identify patients with VH-TCFA in a prospective manner.

## References

[1] Roger VL, Go AS, Lloyd-Jones DM, Benjamin EJ, Berry JD et al. (2012) Heart Disease and Stroke Statistics—2012 Update. Circulation 125: e2-e220. doi:10.1161/CIR.0b013e31823ac046. PubMed: 22179539. 22179539 10.1161/CIR.0b013e31823ac046 PMC4440543

[2] Virmani R, Burke AP, Farb A, Kolodgie FD (2006) Pathology of the Vulnerable Plaque. J Am Coll Cardiol 47: C13-C18. doi:10.1016/j.jacc.2005.10.065. PubMed: 16631505. 16631505 10.1016/j.jacc.2005.10.065

[3] Nair A, Margolis MP, Kuban BD, Vince DG (2007) Automated coronary plaque characterisation with intravascular ultrasound backscatter: ex vivo validation. EuroIntervention 3: 113-120. PubMed: 19737694. 19737694

[4] Naghavi M, Libby P, Falk E, Casscells SW, Litovsky S et al. (2003) From Vulnerable Plaque to Vulnerable Patient: A Call for New Definitions and Risk Assessment Strategies: Part II. Circulation 108: 1772-1778. doi:10.1161/01.CIR.0000087481.55887.C9. PubMed: 14557340. 14557340 10.1161/01.CIR.0000087481.55887.C9

[5] Fleg JL, Stone GW, Fayad ZA, Granada JF, Hatsukami TS et al. (2012) Detection of High-Risk Atherosclerotic Plaque: Report of the NHLBI Working Group on Current Status and Future Directions. J Am Coll Cardiol Imaging 5: 941-955. doi:10.1016/j.jcmg.2012.07.007. 10.1016/j.jcmg.2012.07.007 PMC3646061 22974808

[6] Nair A, Kuban BD, Tuzcu EM, Schoenhagen P, Nissen SE et al. (2002) Coronary Plaque Classification With Intravascular Ultrasound Radiofrequency Data Analysis. Circulation 106: 2200-2206. doi:10.1161/01.CIR.0000035654.18341.5E. PubMed: 12390948. 12390948 10.1161/01.cir.0000035654.18341.5e

[7] Stone GW, Maehara A, Lansky AJ, de Bruyne B, Cristea E et al. (2011) A Prospective Natural-History Study of Coronary Atherosclerosis. N Engl J Med 364: 226-235. doi:10.1056/NEJMoa1002358. PubMed: 21247313. 21247313 10.1056/NEJMoa1002358

[8] Hansson GK (2005) Inflammation, Atherosclerosis, and Coronary Artery Disease. N Engl J Med 352: 1685-1695. doi:10.1056/NEJMra043430. PubMed: 15843671. 15843671 10.1056/NEJMra043430

[9] The Emerging Risk Factors Collaboration (2012) C-Reactive Protein, Fibrinogen, and Cardiovascular Disease Prediction. N Engl J Med 367 (1310-1320) 10.1056/NEJMoa1107477 PMC3714101 23034020

[10] Charo IF, Ransohoff RM (2006) The Many Roles of Chemokines and Chemokine Receptors in Inflammation. N Engl J Med 354: 610-621. doi:10.1056/NEJMra052723. PubMed: 16467548. 16467548 10.1056/NEJMra052723

[11] Zernecke A, Shagdarsuren E, Weber C (2008) Chemokines in Atherosclerosis: An Update. Arterioscler Thromb Vasc Biol 28: 1897-1908. doi:10.1161/ATVBAHA.107.161174. PubMed: 18566299. 18566299 10.1161/ATVBAHA.107.161174

[12] Nelken NA, Coughlin SR, Gordon D, Wilcox JN (1991) Monocyte chemoattractant protein-1 in human atheromatous plaques. J Clin Invest 88: 1121-1127. doi:10.1172/JCI115411. PubMed: 1843454. 1843454 10.1172/JCI115411 PMC295565

[13] Yu X, Dluz S, Graves DT, Zhang L, Antoniades HN et al. (1992) Elevated expression of monocyte chemoattractant protein 1 by vascular smooth muscle cells in hypercholesterolemic primates. Proc Natl Acad Sci U S A 89: 6953-6957. doi:10.1073/pnas.89.15.6953. PubMed: 1379728. 1379728 10.1073/pnas.89.15.6953 PMC49623

[14] Poddar R, Sivasubramanian N, DiBello PM, Robinson K, Jacobsen DW (2001) Homocysteine Induces Expression and Secretion of Monocyte Chemoattractant Protein-1 and Interleukin-8 in Human Aortic Endothelial Cells : Implications for Vascular Disease. Circulation 103: 2717-2723. doi:10.1161/01.CIR.103.22.2717. PubMed: 11390343. 11390343 10.1161/01.cir.103.22.2717

[15] Li M, Chen J, Li Y-S, Feng Y-B, Zeng Q-T (2007) Folic acid reduces chemokine MCP-1 release and expression in rats with hyperhomocystinemia. Cardiovasc Pathol 16: 305-309. doi:10.1016/j.carpath.2007.03.005. PubMed: 17868882. 17868882 10.1016/j.carpath.2007.03.005

[16] Solini A, Santini E, Ferrannini E (2006) Effect of short-term folic acid supplementation on insulin sensitivity and inflammatory markers in overweight subjects. Int J Obes 30: 1197-1202. doi:10.1038/sj.ijo.0803265. PubMed: 16491109. 10.1038/sj.ijo.0803265 16491109

[17] Economou EV, Malamitsi-Puchner AV, Pitsavos CP, Kouskouni EE, Magaziotou-Elefsinioti I et al. (2004) Negative Association between Circulating Total Homocysteine and Proinflammatory Chemokines MCP-1 and RANTES in Prepubertal Lean, but Not in Obese, Children. J Cardiovasc Pharmacol 44: 310-315. doi:10.1097/01.fjc.0000133587.01718.59. PubMed: 15475827. 15475827 10.1097/01.fjc.0000133587.01718.59

[18] Nygård O, Vollset SE, Refsum H, Brattström L, Ueland PM (1999) Total homocysteine and cardiovascular disease. J Intern Med 246: 425-454. doi:10.1046/j.1365-2796.1999.00512.x. PubMed: 10583714. 10583714 10.1046/j.1365-2796.1999.00512.x

[19] Kaul S, Zadeh AA, Shah PK (2006) Homocysteine Hypothesis for Atherothrombotic Cardiovascular Disease: Not Validated. J Am Coll Cardiol 48: 914-923. doi:10.1016/j.jacc.2006.04.086. PubMed: 16949480. 16949480 10.1016/j.jacc.2006.04.086

[20] Antoniades C, Antonopoulos AS, Tousoulis D, Marinou K, Stefanadis C (2009) Homocysteine and coronary atherosclerosis: from folate fortification to the recent clinical trials. Eur Heart J 30: 6-15. PubMed: 19029125. 19029125 10.1093/eurheartj/ehn515

[21] Hoffmann M (2001) Hyperhomocysteinemia enhances vascular inflammation and accelerates atherosclerosis in a murine model. J Clin Invest 107: 675-683. doi:10.1172/JCI10588. PubMed: 11254667. 11254667 10.1172/JCI10588 PMC208940

[22] Joseph J, Handy DE, Loscalzo J (2009) Quo Vadis: Whither Homocysteine Research? Cardiovasc Toxicol 9: 53-63. doi:10.1007/s12012-009-9042-6. PubMed: 19484390. 19484390 10.1007/s12012-009-9042-6 PMC3266720

[23] Baigent C, Clarke R (2007) B Vitamins for the Prevention of Vascular Disease: Insufficient Evidence to Justify Treatment. JAMA 298: 1212-1214. doi:10.1001/jama.298.10.1212. PubMed: 17848657. 17848657 10.1001/jama.298.10.1212

[24] Ebbing M, Bleie O, Ueland PM, Nordrehaug JE, Nilsen DW et al. (2008) Mortality and Cardiovascular Events in Patients Treated With Homocysteine-Lowering B Vitamins After Coronary Angiography: A Randomized Controlled Trial. JAMA 300: 795-804. doi:10.1001/jama.300.7.795. PubMed: 18714059. 18714059 10.1001/jama.300.7.795

[25] Ebbing M, Bønaa KH, Arnesen E, Ueland PM, Nordrehaug JE et al. (2010) Combined analyses and extended follow-up of two randomized controlled homocysteine-lowering B-vitamin trials. J Intern Med 268: 367-382. doi:10.1111/j.1365-2796.2010.02259.x. PubMed: 20698927. 20698927 10.1111/j.1365-2796.2010.02259.x

[26] Løland KH, Bleie Ø, Blix AJ, Strand E, Ueland PM et al. (2010) Effect of Homocysteine-Lowering B Vitamin Treatment on Angiographic Progression of Coronary Artery Disease: A Western Norway B Vitamin Intervention Trial (WENBIT) Substudy. Am J Cardiol 105: 1577-1584. doi:10.1016/j.amjcard.2010.01.019. PubMed: 20494665. 20494665 10.1016/j.amjcard.2010.01.019

[27] Ebbing M, Bleie Ø, Ueland PM, Nordrehaug JE, Nilsen DW et al. (2008) Mortality and cardiovascular events in patients treated with homocysteine-lowering B vitamins after coronary angiography: a randomized controlled trial. JAMA 300: 795-804. doi:10.1001/jama.300.7.795. PubMed: 18714059. 18714059 10.1001/jama.300.7.795

[28] Bleie Ø, Strand E, Ueland PM, Vollset SE, Refsum H et al. (2011) Coronary blood flow in patients with stable coronary artery disease treated long term with folic acid and vitamin B12. Coron Artery Dis 22: 270-278. doi:10.1097/MCA.0b013e328344fff4. PubMed: 21389855. 21389855 10.1097/MCA.0b013e328344fff4

[29] Nasu K, Tsuchikane E, Katoh O, Vince DG, Virmani R et al. (2006) Accuracy of In Vivo Coronary Plaque Morphology Assessment: A Validation Study of In Vivo Virtual Histology Compared With In Vitro Histopathology. J Am Coll Cardiol 47: 2405-2412. doi:10.1016/j.jacc.2006.02.044. PubMed: 16781367. 16781367 10.1016/j.jacc.2006.02.044

[30] García-García HM, Mintz GS, Lerman A, Vince DG, Margolis MP et al. (2009) Tissue characterisation using intravascular radiofrequency data analysis: recommendations for acquisition, analysis, interpretation and reporting. EuroIntervention 5: 177-189. doi:10.4244/EIJV5I2A29. PubMed: 20449928. 20449928 10.4244/eijv5i2a29

[31] R Development Core Team (2012) R: A Language and Environment for Statistical Computing. 2.15.0 ed. Vienna, Austria: R Foundation for Statistical Computing.

[32] Honaker J, King G, Blackwell M (2010) Amelia: Amelia II: A Program for Missing Data. R Package Version 1: 2-18 ed

[33] Kim S-H, Lee J-W, Im J-A, Hwang H-J (2011) Monocyte chemoattractant protein-1 is related to metabolic syndrome and homocysteine in subjects without clinically significant atherosclerotic cardiovascular disease. Scand J Clin Lab Invest 71: 1-6. doi:10.3109/00365513.2010.519047. PubMed: 21073393. 21073393 10.3109/00365513.2010.519047

[34] Deo R, Khera A, McGuire DK, Murphy SA, de P Meo Neto J, et al. (2004) Association among plasma levels of monocyte chemoattractant protein-1, traditional cardiovascular risk factors, and subclinical atherosclerosis. J Am Coll Cardiol 44: 1812-1818. 15519012 10.1016/j.jacc.2004.07.047

[35] Piemonti L, Calori G, Lattuada G, Mercalli A, Ragogna F et al. (2009) Association Between Plasma Monocyte Chemoattractant Protein-1 Concentration and Cardiovascular Disease Mortality in Middle-Aged Diabetic and Nondiabetic Individuals. Diabetes Care 32: 2105-2110. doi:10.2337/dc09-0763. PubMed: 19641159. 19641159 10.2337/dc09-0763 PMC2768199

[36] de Lemos JA, Morrow DA, Sabatine MS, Murphy SA, Gibson CM et al. (2003) Association Between Plasma Levels of Monocyte Chemoattractant Protein-1 and Long-Term Clinical Outcomes in Patients With Acute Coronary Syndromes. Circulation 107: 690-695. doi:10.1161/01.CIR.0000049742.68848.99. PubMed: 12578870. 12578870 10.1161/01.cir.0000049742.68848.99

[37] de Lemos JA, Morrow DA, Blazing MA, Jarolim P, Wiviott SD et al. (2007) Serial Measurement of Monocyte Chemoattractant Protein-1 After Acute Coronary Syndromes: Results From the A to Z Trial. J Am Coll Cardiol 50: 2117-2124. doi:10.1016/j.jacc.2007.06.057. PubMed: 18036447. 18036447 10.1016/j.jacc.2007.06.057

[38] Ortlepp JR, Vesper K, Mevissen V, Schmitz F, Janssens U et al. (2003) Chemokine receptor (CCR2) genotype is associated with myocardial infarction and heart failure in patients under 65 years of age. J Mol Med (Berl) 81: 363-367. PubMed: 12719858. 12719858 10.1007/s00109-003-0435-x

[39] Hong M-K, Mintz GS, Lee CW, Lee J-W, Park J-H et al. (2008) A Three-Vessel Virtual Histology Intravascular Ultrasound Analysis of Frequency and Distribution of Thin-Cap Fibroatheromas in Patients With Acute Coronary Syndrome or Stable Angina Pectoris. J Am Coll Cardiol 101: 568-572. doi:10.1016/j.amjcard.2007.09.113. 10.1016/j.amjcard.2007.09.113 18308000

[40] Sanidas EA, Maehara A, Mintz GS, Kashiyama T, Guo J et al. (2011) Angioscopic and Virtual Histology Intravascular Ultrasound Characteristics of Culprit Lesion Morphology Underlying Coronary Artery Thrombosis. Am J Cardiol 107: 1285-1290. doi:10.1016/j.amjcard.2010.12.037. PubMed: 21414594. 21414594 10.1016/j.amjcard.2010.12.037

[41] Virmani R, Kolodgie FD, Burke AP, Farb A, Schwartz SM (2000) Lessons From Sudden Coronary Death : A Comprehensive Morphological Classification Scheme for Atherosclerotic Lesions. Arterioscler Thromb Vasc Biol 20: 1262-1275. doi:10.1161/01.ATV.20.5.1262. PubMed: 10807742. 10807742 10.1161/01.atv.20.5.1262

[42] Li J, Guo Y, Luan X, Qi T, Li D et al. (2012) Independent Roles of Monocyte Chemoattractant Protein-1, Regulated on Activation, Normal T-Cell Expressed and Secreted and Fractalkine in the Vulnerability of Coronary Atherosclerotic Plaques. Circ J 76: 2167-2173. doi:10.1253/circj.CJ-11-1457. PubMed: 22664781. 22664781 10.1253/circj.cj-11-1457

[43] Gerszten RE, Tager AM (2012) The Monocyte in Atherosclerosis - Should I Stay or Should I Go Now? N Engl J Med 366: 1734-1736. doi:10.1056/NEJMcibr1200164. PubMed: 22551134. 22551134 10.1056/NEJMcibr1200164

[44] Libby P (2013) Mechanisms of Acute Coronary Syndromes and Their Implications for Therapy. N Engl J Med 368: 2004-2013. doi:10.1056/NEJMra1216063. PubMed: 23697515. 23697515 10.1056/NEJMra1216063

[45] Cheruvu PK, Finn AV, Gardner C, Caplan J, Goldstein J et al. (2007) Frequency and Distribution of Thin-Cap Fibroatheroma and Ruptured Plaques in Human Coronary Arteries: A Pathologic Study. J Am Coll Cardiol 50: 940-949. doi:10.1016/j.jacc.2007.04.086. PubMed: 17765120. 17765120 10.1016/j.jacc.2007.04.086

